# Supplementary material for: Temperature-Dependent Excitonic Light Manipulation with Atomically Thin Optical Elements
Source: Nano Lett. 2024 Apr 5;24(21):6240–6. doi: 10.1021/acs.nanolett.4c00694 (PMC11140734; doi:10.1021/acs.nanolett.4c00694)
Supplement: Supplementary file 1 — nl4c00694_si_001.pdf [file nl4c00694_si_001.pdf]

Supporting Information for: **Temperature-dependent excitonic light manipulation with atomically-thin optical elements**

Ludovica Guarneri[1], Qitong Li[2], Thomas Bauer[1], Jung-Hwan Song[2], Ashley P. Saunders[3], Fang Liu[3], Mark L. Brongersma[2*], and Jorik van de Groep[1*]

[1] *Van der Waals-Zeeman Institute, Institute of Physics, University of Amsterdam, Amsterdam, 1098 XH, the Netherlands*
[2] *Geballe Laboratory for Advanced Materials, Stanford University, Stanford, CA 94305, USA*
[3] *Department of Chemistry, Stanford University, Stanford, CA 94305, USA*

[*] brongersma@stanford.edu; j.vandegroep@uva.nl

Methods

Large-area exfoliation: The WS_2 monolayers are exfoliated onto the sapphire substrate from bulk WS_2 single crystals (HQ graphene) using the gold tape exfoliation method. In brief, a 100 nm Au layer is evaporated onto a clean Si wafer (Nova wafers) and is subsequently coated with Polyvinylpyrrolidone (PVP) to protect against contaminations. A piece of thermal release tape (Nitto, RA95LS) is used to template strip the Au layer off the Si substrate. The Au surface is immediately brought into contact with a freshly cleaved WS_2 crystal surface, and subsequently peels off a monolayer. The tape/PVP/Au/monolayer stack is stamped onto the sapphire substrate. The thermal release tape is removed by heat, and the PVP protection layer is dissolved in water. The Au layer is removed by a mild gold etchant solution (KI/I_2), which reacts with gold but leaves monolayers intact. After rinsing with water and isopropanol, a clean WS_2 monolayer is obtained on the sapphire substrate.

Sample fabrication: We start the fabrication by patterning a series of global gold markers around the exfoliated WS_2 monolayer to locate the homogeneous monolayer areas on the substrate, using a procedure described in ref. [14]. After determining the positions to nanopattern the atomically thin lenses with an optical microscope, a methyl methacrylate (MMA)/polymethyl methacrylate (PMMA) double-layer is spin coated to serve as a positive-tone electron-beam resist layer. A thin conductive polymer layer (E-Spacer 300Z) is further spin coated to mitigate charging effects during the electron-beam lithography process (JEOL 6300 100 kV system, electron-beam current = 4.5 nA, dose = 500 $\mu C\ cm^{-2}$). After the development in a methyl isobutyl ketone:isopropyl alcohol (1:1) solution for 45 s, we perform gentle (RF power = 30 W) argon plasma etching (Oxford PlasmaPro 80) for 20 s to only etch the WS_2 monolayer WS_2 away. The remaining resist is finally removed by rinsing the sample in acetone overnight.

Spectroscopy: Raman, photoluminescence (PL), and reflection spectroscopy on the zone plate lens are performed in a Witec α300R confocal raman microscope with a 532 nm fiber-coupled diode laser as an excitation source for the Raman and PL measurements and a halogen lamp for the reflectance measurements. For the Raman and PL measurements, a 100× Zeiss EC Epiplan-Neofluar (NA = 0.9) microscope objective is used to collect from a diffraction-limited area on the sample. For the reflectance measurements, a 50× Zeiss EC Epiplan (NA = 0.75) microscope objective is used, where we close the aperture stop of the Köhler illumination to minimize the angular range of the incident light and normalize the spectra to the reflectance of the sapphire substrate. For all experiments a photonic-crystal fiber (7 µm effective core diameter) is used to send the collected signal to a Witec UHTS300 SMFC VIS spectrograph (150 lines/mm grating for reflectance, 600 lines/mm grating for PL, 1800 lines/mm grating for Raman) coupled to a Andor Newton EMCCD camera cooled to -60 ℃ to suppress electronic noise.

Room-temperature focusing experiments: See Fig. S2 for a schematic representation of the setup. A supercontinuum laser (NKT SuperK extreme EXW-12) coupled to an acousto-optical tunable filter (AOTF,

SuperK select, ~5 nm linewidth) is used to control the intensity and wavelength of the incident light. The sample is scanned with respect to the incident light inside the same Witec α300R microscope, using a 3D piezo stage. A 100× Zeiss EC Epiplan-Neofluar (NA = 0.9) microscope objective is used to collect the local light intensity which is then coupled into a 25-µm fiber. Finally, the signal is detected by an avalanche photodiode (APD) using an integration time of 0.01 s per pixel.

Cryogenic experiments: See Fig. 4b for a schematic representation of the setup. For the focusing experiments, laser light from the same supercontinuum laser and AOTF is fiber coupled and used to illuminate the sample, inside the cryostat (Oxford Instruments Optistat AC-V12). A f = 75 mm lens (Thorlabs AC254-A-ML) combined with a f = 500 mm tube lens (Thorlabs AC254-A-ML) is used to image the zone-plate's focus onto a CMOS camera (IDS, U3-3880CP-M-GL, Rev.2.2) with a magnification of M = 6.7. For reflectance measurements a Thorlabs OSL2 halogen lamp is used to illuminate the sample with unpolarized light. The reflected signal is collected by the same f = 75 mm lens, after which it is coupled into a collection fiber (core diameter: 105 µm; Thorlabs M15L20) using an f = 80 mm lens (Thorlabs AC254-A-ML) and sent to a Princeton Instruments SpectraPro 2300i spectrometer (300 lines/mm) and a Pixis Si charge-coupled device (CCD; −120 °C detector temperature).

Section 1 - Zone plate lens design

The zone plate lens is designed using the following target parameters:

- focal length f = 1 mm
- design optimized for λ = 615 nm
- diameter d = 500 μm
- within the 250 μm radius of the lens, there are a total of 102 concentric rings including the central region (i.e. central region is considered ring i = 1).

The outer radius of ring i is given by: $r_i^{max} = \sqrt{i\,\lambda(f + i\,\lambda/4)}$. In the fabricated lens, the odd rings were covered by monolayer WS_2, while the monolayer was etched in the even rings to expose the underlying sapphire substrate.

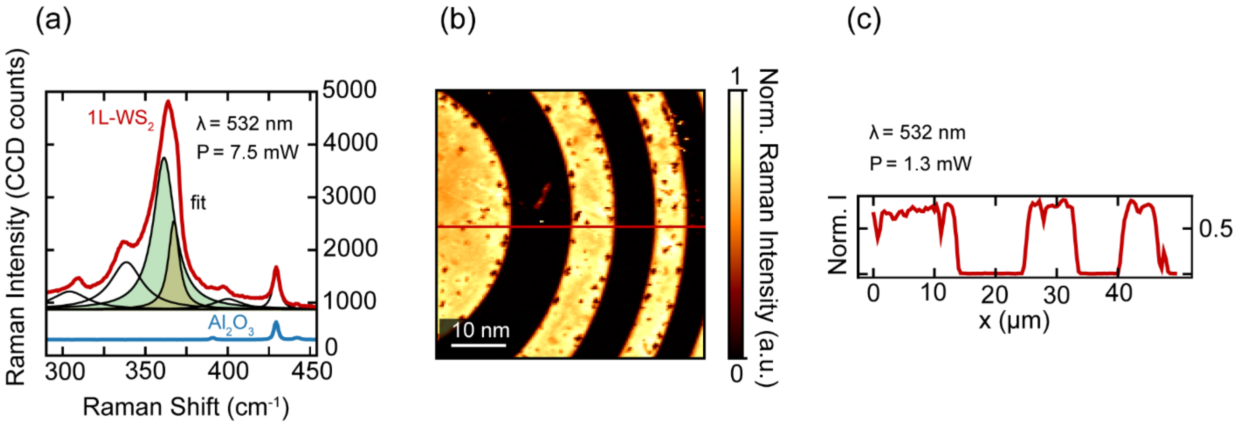

Figure S1: Raman characterization of the lens. (a) Raman spectrum of monolayer WS_2 (red solid line), with Lorentzian fitting of the relevant Raman modes (black solid lines), and Raman spectrum of the Al_2O_3 substrate (blue solid line). (b) Spatially resolved intensity map of the sum of the two main Raman modes of WS_2 integrated over their width, 2LA(M) (shaded green in a) and $E_{2g}^1(\Gamma)$ (shaded orange in a). (c) Spatial line-scan along part of the zone plate radius (red line in b) of the same integrated intensity, confirming the patterning of the WS_2.

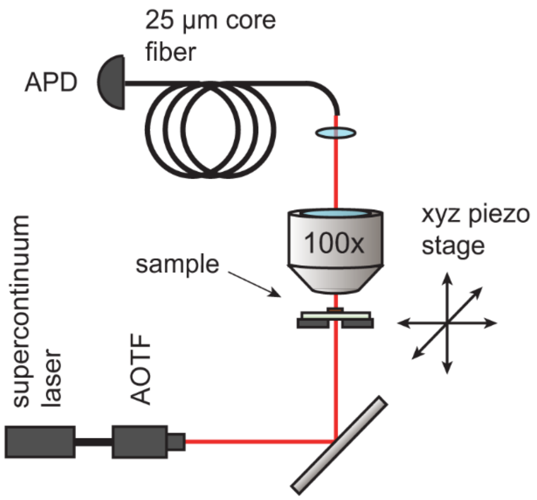

Figure S2: Schematic of confocal microscope setup used for room temperature focusing efficiency measurements. AOTF: acousto-optic tunable filter, APD: avalanche photodiode.

Section 2 - Focusing efficiency fitting procedure

To retrieve the focusing efficiency $\eta = P_{focus}/P_{incident}$, we fit both the room temperature (Fig. 2) and the temperature-dependent (Fig. 4a) data using the same protocol. The power in the focus (P_{focus}) is obtained from the intensity map (or image for the temperature-dependent measurements) of the focus in the *xy*-plane at the focal height z = *f* (Fig. 2b). As an example, Fig. S3 shows the x- and y-crosscuts of the focus measured in the confocal configuration at 620 nm (Fig. 2b). We fit the data using an Airy function *g*:

$$g = |b_{inc}|^2 + \left|A \cdot \frac{J_1(r)}{r} - b_{coh}\right|^2. \qquad \text{(S2-1)}$$

Here, *A* is the amplitude of the focus peak, J_1 is the Bessel function of the first kind. The sum $|b_{coh}|^2 + |b_{inc}|^2$ represents the intensity incident on the lens, where b_{coh} is the coherent contribution and contains all additional scattering events within the coherence length of the exciton, while b_{inc} is the incoherent contribution stemming from all light not interacting with the lens. Lastly, *r* is defined as:

$$r = k \cdot \sqrt{(x - x_o)^2 + (y - y_o)^2} \cdot \mathrm{NA}_{eff}\ , \qquad \text{(S2-2)}$$

where $k = \frac{2\pi}{\lambda}$ is the wavevector, x_o, y_o are the peak centers of the x and y cross-sections, respectively, and NA_{eff} represents the effective numerical aperture of the collection system, taking spherical aberrations and restricting apertures into account.

To retrieve the power in the focus we integrate *g* along x and y in the 2D-range over the central lobe ($[x_2 - x_1; y_2 - y_1]$, minimum to minimum closest to the focal point in Fig. S3). The power incident on the sample surface, denominator of the focusing efficiency, is defined as $P_{incident} = (\,|b_{coh}|^2 + |b_{inc}|^2\,) \cdot A_{lens}$, where A_{lens} is the area of the zone-plate lens.

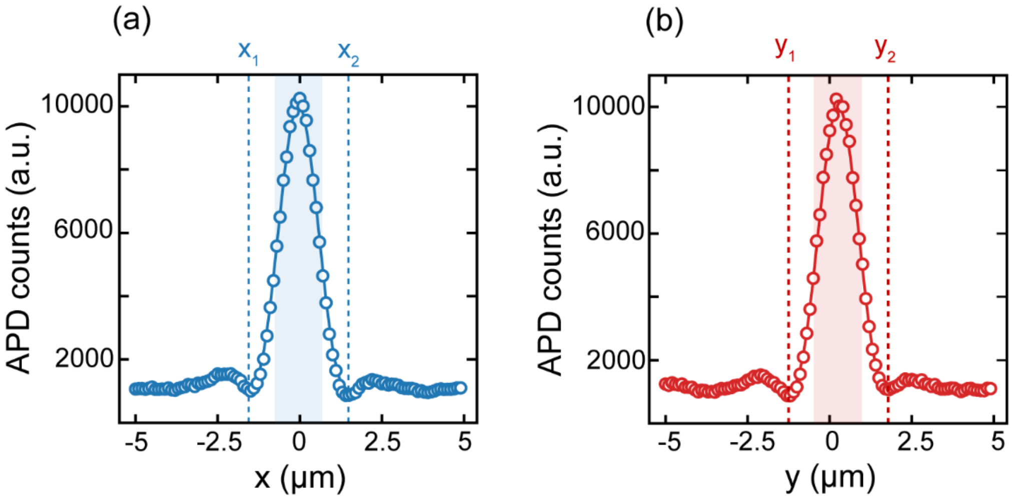

Figure S3: Fitting of the focal shape in the xy-plane. (a) x-cross-section of the field intensity mapped over the lens plane (xy) at the focal height $z = f$ (Fig. 2b). The blue circles show the experimental data points, while the continuous blue line is the fitting according to Eq. S2-1. (b) y-cross-section of the experimental data (red circles) and fitting (red line). The blue (red)-shaded area highlights the FWHM and corresponds to 1.444 µm (1.443 µm), while the minimum-to-minimum distance is $x_2 - x_1 = 3.035\ \mu m$ ($y_2 - y_1 = 3.034\ \mu m$), indicating that the focal spot is diffraction limited when compared to the Rayleigh criterion.

Section 3 – Focusing efficiency error analysis

We estimate the error bars of the focusing efficiency measurements by considering all the relevant sources of uncertainty involved in the measurements. These are: (i) the process of locating the focal point (z-position above the lens surface) at which we measure the field intensity, (ii) uncertainties coming from the fitting procedure and (iii) fluctuations of the laser power.

We find that the choice of the z-position is the dominant source of uncertainty. The error is calculated as the standard deviation of 10 measurements of the focusing efficiency at 630 nm. For these measurements, the focal height is determined by manually adjusting the corresponding z-stage (Thorlabs PT3A/M) with micrometric accuracy. Prior to each measurement, we deliberately move away from the optimized z-position and try to find back the optimal z-coordinate, reproducing the measurement conditions. From the statistical analysis we find an error of 6%. Since the measurements are performed in the same fashion at all temperatures and wavelengths, it is reasonable to assume that the error quantification applies to all the experiemental data points. We set the error bar to be $1\sigma(\lambda) = 0.06 \cdot \eta(\lambda)$ per side, i.e. for each data point the bottom and top of the shadded area around the data point ranges within the interval $[\eta(\lambda) - \sigma(\lambda);\ \eta(\lambda) + \sigma(\lambda)]$.

We assess the error bars for the most stringent experimental conditions in the cryostat setup shown in Figure 4b and at room temperature. We then use this upper limit error also for the room temperature data measured using the setup shown in S2. Here, we use the same laser and fitting procedure, while the z-positioning is selected with higher accuracy (here, the focal height is selected with a motorized-stage and using the x-z intensity map to find the brightest pixel). We have also tested the fluctuations of the temperature from the targeted one. The natural room temperature of our laboratory is 294 K, with negligible fluctuations. For the other three temperatures we report (197.958 ± 0.094) K, (99.785 ± 0.983) K and (13.355 ± 0.082) K. The temperature fluctuation are in all cases < 1 K and thus we do not consider temperature fluctuations as a source of error in the focusing efficiency.

Section 4 - Theoretical evaluation of zone plate lens efficiency

To provide a comparison to the focusing efficiency at room temperature, we use diffraction theory to obtain a theoretical prediction of the efficiency of our atomically-thin lens. First, we use the optical constants derived from the fit to the reflection spectrum (see Eq. 1 in the main manuscript and supporting information section 5) in a transfer-matrix calculation to find the complex transmission coefficient of the sapphire/WS_2/air layer stack and bare sapphire/air interface. These complex transmission coefficients describe the local amplitude and phase of the scattered fields at the surface of the zone plate lens in the covered and open rings, respectively. Second, we employ scalar Rayleigh-Sommerfeld diffraction theory [1] to calculate the intensity in the focus of the lens. To determine the focusing efficiency, we employ the same fitting routing to the calculated intensity profiles as used for the experimental data. The resulting focusing efficiency spectrum in Fig. S4 shows good correspondence to the experimental focusing efficiency spectrum in Fig. 2c.

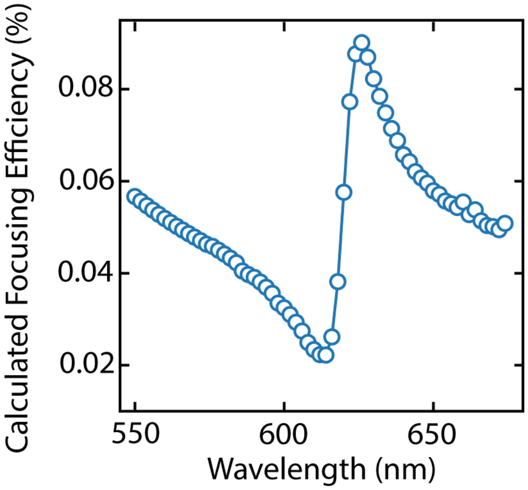

Figure S4: Calculated focusing efficiency of the zone plate lens. Theoretical focusing efficiency of the atomically-thin lens obtained via transfer matrix calculations and diffraction theory. The optical constants of the monolayer used in the calculations are derived from the fit to the reflectance spectrum (see Equation 1 in the main text and supporting information section 5).

Section 5 – Reflectance fitting - Numerical analysis

We apply a Savitzky-Golay filter (window-length = 5, polynomial order =1) to reduce the noise in the experimental reflectance spectra at the edges of the measured spectral range to better facilitate the fitting procedure. Using a transfer-matrix method (TMM), we calculate the reflectance of monolayer WS_2 on sapphire. For the fitting of the temperature dependent reflectance, we account for the contributions of the rear side of the substrate as an incoherent contribution in the TMM. We model the susceptibility with a multi-Lorentzian function and minimizing the residual between the experimental and calculated reflectance. Specifically, we use N = 200-500 equally spaced Lorentz oscillators with width of 10 meV, considering the available experimental energy range. This method, outlined by Li et al.[2], is a fully numerical approach to retrieve the susceptibility and the narrow-band Lorentz oscillators bear no physical interpretation. For the Kramers-Kronig relation to be valid, it is necessary to consider optical transitions outside the experimental range. Below 1.4 eV, no relevant transitions occur that would affect the trends in the range of

interest[2]. To compensate the lack of experimental data above 3 eV we add χ_∞ [3], a constant value accounting for the higher energy electronic transitions.

$$\chi(E) = \chi_\infty + \sum_{j=1}^{N} \frac{f_j}{E_j^2 - E^2 - iE\gamma} \qquad \text{(S5-1)}$$

Here $\chi_\infty = (\epsilon_\infty - 1) = 14$ [4], f_j and E_j are, respectively, the oscillator strength and the central energy of the jth Lorentzian oscillator, $\gamma = 10$ meV is the fixed width of the oscillators.

To confirm the soundness of our method, we first compare the extracted susceptibility to published ellipsometry-measured susceptibility values of a CVD-grown monolayer WS_2 on sapphire[5] (Fig. S5b). For the imaginary part, we find a good match across the whole energy range. For the real part, we observe an unreliably fast drop at high energies approaching the end of the experimental range, where the data becomes noisier. We therefore exclude this portion of the energy range from the subsequent physically-meaningful fit, detailed in the main text and described by equation 1. For the temperature dependent measurements, we find that it becomes essential to include additional oscillators in the physically meaningful fit, which describe the contributions of excited exciton states[2].

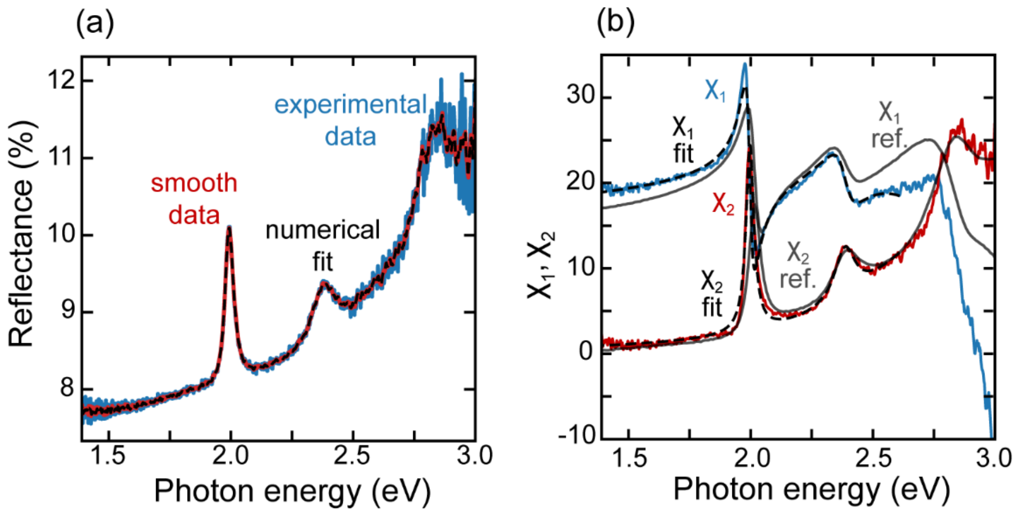

Figure S5: Fitting of the experimental reflectance and electronic susceptibility of monolayer WS_2. (a) Experimental reflectance spectrum of monolayer WS_2 on Al_2O_3 (blue solid line), smoothened data (red solid line) and numerical fitting (dashed black line). (b) Real part (χ_1, blue solid line) and imaginary part (χ_2, red solid line) of the complex susceptibility of monolayer WS_2 retrieved from the numerical fitting, physically meaningful fit (black dashed line) using the model described in the main text in Eq. (1), real and imaginary part of the susceptibility re-plotted from ref.[5] (grey solid lines).

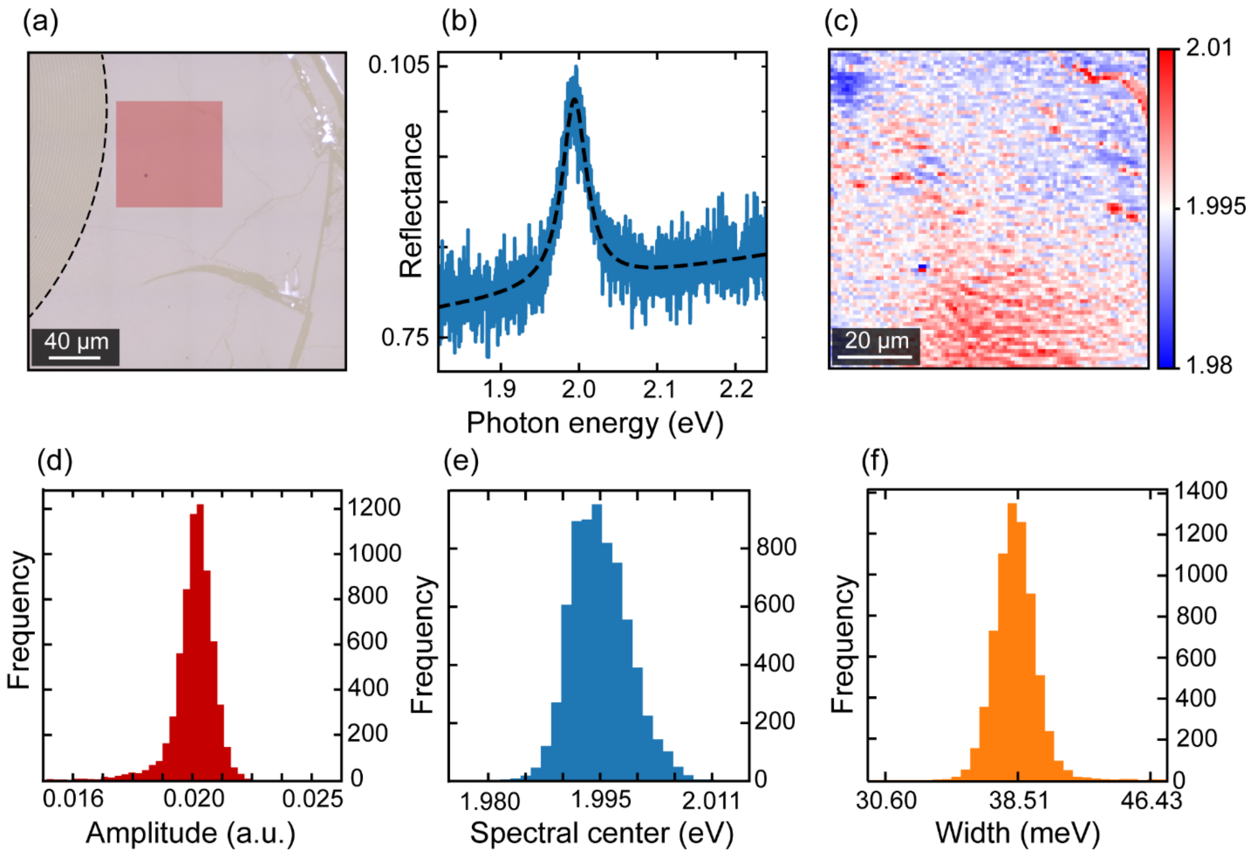

Figure S6: Quantification of the spectral broadening caused by inhomogeneities in the monolayer. (a) Optical microscope image of the sample. The dashed black line highlights the lens contour. The red shaded square indicates the region mapped in c), chosen to have the same area and location as the region probed to obtain the reflectance spectra in Fig. 4c. (b) Exemplary reflectance spectrum (blue line) measured within the red shaded region highlighted in a) and fitting (black dashed line). The fitting function comprises a Lorentzian function and a polynomial (constant + First order term). (c) Spectral center of the exciton resonance in reflectance mapped across the red shaded area in a). The different spectral location of the excitonic peak across the measured region is one of the sources of spectral broadening. (d, e, and f) Histograms illustrating the spread of the exciton amplitude (red), spectral center (blue) and width (orange). The histograms are obtained by fitting each reflectance spectrum in the shaded red area shown in a) in the same fashion as illustrated in b). Amplitude, spectral center, and width correspond to those of the Lorentzian function. The spectral broadening of 5.045 meV corresponds to one standard deviation of the spectral center shown in e).

References

1. Marathay, A. S. & Mccalmont, J. F. On the usual approximation used in the Rayleigh-Sommerfeld diffraction theory. *J. Opt. Soc. Am. A* **21**, 510–516 (2004).
2. Li, Y. *et al.* Measurement of the optical dielectric function of monolayer transition-metal dichalcogenides: MoS_2,$MoSe_2$,WS_2 and WSe_2. *Phys Rev B* **90**, 205422 (2014).
3. Li, M., Hail, C. U., Biswas, S. & Atwater, H. A. Excitonic Beam Steering in an Active van der Waals Metasurface. *Nano Lett* **23**, 2771–2777 (2023).
4. Laturia, A., Van de Put, M. L. & Vandenberghe, W. G. Dielectric properties of hexagonal boron nitride and transition metal dichalcogenides: from monolayer to bulk. *NPJ 2D Mater Appl* **2**, 6 (2018).
5. Jung, G. H., Yoo, S. J. & Park, Q. H. Measuring the optical permittivity of two dimensional materials without a priori knowledge of electronic transitions. *Nanophotonics* **8**, 263–270 (2018).
